# The intestine microbiota of shrimp and its impact on cultivation

**DOI:** 10.1007/s00253-024-13213-3

**Published:** 2024-06-06

**Authors:** Shenzheng Zeng, Jianguo He, Zhijian Huang

**Affiliations:** 1 State Key Laboratory of Biocontrol, School of Marine Sciences, Sun Yat-Sen University, Guangzhou, People’s Republic of China; 2 China-ASEAN Belt and Road Joint Laboratory On Mariculture Technology, Southern Marine Sciences and Engineering Guangdong Laboratory (Zhuhai), Zhuhai, People’s Republic of China

**Keywords:** Shrimp intestine microbiota, Microecological Koch’s postulates, Environment–intestinal microbiota–host health relationship, Microbial micro-ecological prevention and control strategy, Healthy intestine microbiota

## Abstract

**Abstract:**

Intestinal microbiome contains several times of functional genes compared to the host and mediates the generation of multiple metabolic products, and therefore it is called “second genome” for host. Crustaceans rank second among the largest subphylum of aquaculture animals that are considered potentially satisfy global substantial food and nutrition security, among which the Pacific white shrimp (*Litopenaeus vannamei*) ranks the first in the production. Currently, increasing evidences show that outbreaks of some most devastating diseases in shrimp, including white feces syndrome (WFS) and acute hepatopancreatic necrosis disease (AHPND), are related to intestinal microbiota dysbiosis. Importantly, the intestine microbial composition can be altered by environmental stress, diet, and age. In this review, we overview the progress of intestinal microbiota dysbiosis and WFS or ANPHD in shrimp, and how the microbial composition is altered by external factors. Hence, developing suitable microbial micro-ecological prevention and control strategy to maintain intestinal balance may be a feasible solution to reduce the risk of disease outbreaks. Moreover, we highlight that defining the “healthy intestine microbiota” and evaluating the causality of intestinal microbiota dysbiosis and diseases following the logic of “Microecological Koch’s postulates” should be the key goal in future shrimp intestinal field, which help to guide disease diagnosis and prevent disease outbreaks in shrimp farming.

**Key points:**

*• Intestinal microbiota dysbiosis is relevant to multiple shrimp diseases.*

*• Microecological Koch’s postulates help to evaluate the causality of shrimp diseases.*

## Introduction

Animals have co-evolved with different kinds of microbes, and therefore, they are so-called “super organisms,” which comprise the host itself and its microbiome (Gómez and Balcázar 2008; Scott et al. 2015; Zhu et al. 2010). Intestinal microbiota is implicated as an essential modulating factor in host health, immunity, and growth (Hughes et al. 2020; Kurilshikov et al. 2021; Pasolli et al. 2019). The study of intestinal microbiome started in human researches (Arumugam et al. 2011; Costea et al. 2018; Knights et al. 2014), and later have extend to other fields, such as farming animals and aquaculture animals (Couch et al. 2021; Hicks et al. 2018; Kokou et al. 2019; Li et al. 2015).

Aquaculture animals, also called “Blue Food” (Hicks et al. 2019; Naylor et al. 2021), which are considered to potentially satisfy global substantial food and nutrition security (Crona et al. 2023). Crustaceans rank the second among the largest subphylum, and shrimp is known with the highest market profit (Cicala et al. 2020; Rajeev et al. 2020). The *Penaeidae* family of shrimp comprises multiple species with considerable values, including *Litopenaeus vannamei* (Pacific white shrimp) and *Penaeus monodon* (Cornejo-Granados et al. 2017). In particular, Pacific white shrimp ranks the first among all aquaculture shrimp with the most excellent breeding capacity, which is cultured in more than 38 countries and areas, with global production of 5.1 million tons and valued at over $29 billion in 2022 (FAO 2023). However, the aquaculture industry of Pacific white shrimp has suffered enormous economic losses as a result of serious bacterial disease outbreaks, such as acute hepatopancreatic necrosis disease (AHPND), hepatopancreas necrosis syndrome (HPNS), and white feces syndrome (WFS) (Aranguren et al. 2021; Huang et al. 2016, 2020; Lee et al. 2015; Restrepo et al. 2021). Exploring the etiology of these diseases has gained wide concerns, which will help to understand how disease breakout and how to reduce the risk of disease occurrence. With the deeper exploring on etiology of AHPND and WFS, more and more studies noticed a close relationship between disease occurrence and the dysbiosis of shrimp intestinal microbiota (Hou et al. 2018a; Xiong et al. 2017) (Table 1). The dysbiosis was identified as a loss of diversity and an increased susceptibility to disease (unhealthy shifts in microbial community composition) (Legrand et al. 2019; Wei et al. 2021). Therefore, evaluating the underlying mechanism of how dysbiosis occurs is the primary propose of studies on intestinal microbiota in shrimp in recent years.

**Table 1:** Current studies about the intestinal biomarkers for shrimp diseases

Disease	Biomarkers	Reference
WFS	*Flavobacteriaceae*, *Rhodobacteraceae*, *Vibrionaceae*, *Vibrio*, et al	Xiong et al. (2017)
	*Candidatus* Bacilloplasma, *Paracoccus*, *Lactococcus, Phascolarctobacterium*, et al	Hou et al. (2018a)
	*Vibrio*, *Candidatus*, Bacilloplasma, *Shewanella*, *Gemmobacter*, *Chitinibacter*, et al	Huang et al. (2020)
	*Vibrio*, *Candidatus*, Bacilloplasma, *Shewanella*, *Acinetobacter*	Wang et al. (2020)
	*E. hepatopenaei*, *V. harveyi*, *V. alginolyticus*	Subash et al. (2023)
	*E. hepatopenaei*, *V. parahaemolyticus*	Aranguren et al. (2021)
AHPND	*Vibrionales*, *Vibrionaceae*	Restrepo et al. (2021)
	*Pirellula*, *Blastopirellula*, *Weissella*, *Anderseniella*, et al	Lu et al. (2023)
	*V. anguillarum*	Shen et al. (2021)

In this review, we will overview the current knowledge on shrimp intestinal microbiota and how it mediates shrimp health from an microecological perspective, mainly focusing on its correlation with the most devastating shrimp bacterial diseases (WFS and AHPND). Afterwards, the factors, including diet and environmental stress that shape intestinal microbiota composition are also summarized. Finally, we will discuss how to maintain to prevent disease outbreaks. We do hope the topics discussed herein will facilitate the development of aquaculture practices to reduce the risk of diseases in shrimp farming.

### Intestinal microbiota dysbiosis and disease outbreaks

WFS etiology has been widely concerned. The microsporidian *Enterocytozoon hepatopenaei* was proposed to be the cause of WFS (Ha et al. 2010), while this assertion was not supported by subsequent infection experiment that no signs of WFS can be detected after direct horizontal transmission of *E*. *hepatopenaei* to healthy shrimp (Tangprasittipap et al. 2013). Later, increasing evidence pointed that intestinal microbiota dysbiosis is related to WFS occurrence by comparing healthy and WFS shrimp. A longitudinal study was conducted to evaluate the disease-discriminatory bacterial taxa were identified by applying random forests regression, which found that *Vibrio*, *Rhodobacteraceae*, and *Flavobacteriaceae* is the biomarker for WFS shrimp (Xiong et al. 2017). Moreover, a microbiota-for-age Z (MAZ) score is proposed based on the compositional changes in different culture period, which is relatively stable among healthy shrimp, but it will dramatically decrease if WFS occurs (Xiong et al. 2017). Similarly, composition and functions of intestinal microbiota were markedly shifted by WFS, as evidenced by significantly higher abundance of *Candidatus Bacilloplasma*, and *Phascolarctobacterium* in WFS shrimp, while the abundance of *Paracoccus* and *Lactococcus* show an opposite trend (Hou et al. 2018a). Besides studies of bacteria, some studies indicate that WFS may be the results of *E*. *hepatopenaei* infection along with *Vibrio parahaemolyticus*, *V. harveyi*, and *V*. *alginolyticus* (Aranguren et al. 2021; Subash et al. 2023).

A bit different from WFS, AHPND is proved to be attributed to the infection of pathogen *V. parahaemolyticus* carrying pVA1 plasmid that expresses deadly toxins PirA^VP^ and PirB (Lee et al. 2015). The plasmid is firstly identified in *V. parahaemolyticus*, and it is found in other *Vibrio* species, such as *V. harveyi* and *V*. *alginolyticus* in following investigations (Lu et al. 2023; Restrepo et al. 2021; Shen et al. 2021), which suggest that AHPND is not caused by only one specific pathogen. The AHPND-infected shrimp showed a lower microbial alpha diversity compared to health shrimp, and the AHPND severity is relevant to the decrease of microbial diversity and stability (Cornejo-Granados et al. 2017; Restrepo et al. 2021). However, even AHPND is caused by pVA1 plasmid, the infection and colonization of *Vibrio* is limited to the microbial diversity or stability of shrimp intestine. For example, increasing the relative abundances of beneficial bacterial microbes (*Paracoccus*, *Ruegeria*, and *Microbacterium*) and stability of bacterial community help to improve *Vibrio* resistance (Guo et al. 2023). Similar study also describes probiotic bacteria is capable of controlling AHPND-causing *Vibrio* populations in shrimp intestine, which is supported that supplement of probiotic bacteria *V*. *diabolicus* increases intestinal microbial diversity and stimulates shrimp survival under pVA1-*V. parahaemolyticus* challenge (Restrepo et al. 2021). Theoretically, the colonization resistance may be attributed to the presence of “gatekeepers,” which show intensive interactions of a few members in the microbial network (Dai et al. 2018; Xiong et al. 2018). The stability of microbial community is largely affected by the presence of gatekeepers because their losses disproportionately endanger the metacommunity (Dai et al. 2019).

### Evaluate the causality of microbial dysbiosis and disease is the key study in future

All the above studies show a close relationship between intestinal microbiota dysbiosis and disease outbreaks. However, is intestinal microbiota dysbiosis the cause or the consequence of disease? Traditionally, Koch’s postulates are a set of guidelines used in microbiology to establish a causative relationship between a specific microorganism and a disease (Grimes 2006; Sultana et al. 2017). However, Koch’s postulates are not suitably used in some complex diseases that might link to more than one pathogen (Singh et al. 2016; Sultana et al. 2017; Vonaesch et al. 2018). Available studies indicate that multiple bacteria, rather than an isolated pathogen, are involved in WFS, and inoculation a single bacterium is not responsible for WFS. Following the logic of Koch’s postulates, using intestinal microbiota transplantation, the dysbiotic intestinal microbiota in recipients (healthy shrimp) can be retrieved from transplantation from WFS shrimp, thereby leading to similar symptom as in WFS shrimp (Huang et al. 2020). Therefore, a fulfilled Koch’s postulates, “Microecological Koch’s postulates,” is proposed to explain the etiology of WFS. Similarly, along with development of high-throughput sequencing technology, microbiome approaches evaluate more comprehensive information about disease-related microbes, which help to guide the selection of target bacteria for regression infection. Collectively, as studies of the relationship between intestinal microbiota and shrimp health are increasing, evaluating the causative agents may be the key goal in future study. Clear understanding what the causative agents are will help with subsequent disease treatment and prevention. On the basis of diagnosing the cause of the disease, establishing a more stable and diverse intestinal community may be a potential approach to decrease the risk of disease outbreaks. For example, prebiotics and probiotics are essential to prevent diseases in cultured shrimps by fostering microbiota biodiversity and increasing the abundance of beneficial microbes, which means gathering all beneficial microbes as a beneficial “meta-community” help to treat shrimp diseases (Ochoa-Romo et al. 2022).

### Environmental factors and microbial communities shaping intestinal microbiota

The intestinal microbiota of shrimp will be adjusted by water physical–chemical parameters (ammonia nitrogen, water temperature, salinity, pH), so that the dynamic balance of intestinal microbiota and its symbiotic state with the host are more conducive to adapt to the changes of the external environment (Duan et al. 2019; Zhao et al. 2018).The intestinal microbiota of shrimp is closely related to the salinity of the water, which shows that the relative abundance of opportunistic pathogens (e.g., *Vibrio*) is higher under high salinity (Hou et al. 2020). Moreover, intestinal microbiota of shrimp in high salinity exhibits less connected and lower competitive intestine bacterial interspecies interactions, which indicates that high salinity may lead to host at a higher risk of disease. Similar study shows that there is a significant correlation between the growth characteristics, intestinal microbiota, and water salinity; yet, as salinity gets higher, the abundance of opportunistic pathogens increases, while the number of beneficial bacteria decreases, indicating that the change of salinity will lead to the intestinal composition shifts, even may cause intestinal dysbiosis (Zhang et al. 2016). Studies on *Penaeus monodon* shows that the abundance of *Vibrio* in the high salinity group is higher than that in the low salinity group, while the abundance of *Shewanella* shows an opposite trend (Chaiyapechara et al. 2021). Therefore, in order to effectively construct the structure of shrimp healthy intestinal microbiota, the salinity level of aquaculture water needs to be considered.

As an important environmental condition of aquaculture, water temperature can also affect the composition, structure, and dynamic balance of intestinal microbiota. Low temperature stress reduces the relative abundance of *Bacteroidetes*, *Bacteroidia*, and *Alphaproteobacteria* in shrimp intestine, and the expression of TLR, IMD, proPO, and Casp3 significantly decrease, which suggests cold stress may reduce shrimp immunity to pathogens and antibacterial activity and lead to the reduction of resistance of shrimp to pathogens (Wang et al. 2020). Both temperature and ammonia nitrogen stress could significantly change the structure of intestinal microbiota, resulting in an increase in the abundance of *Firmicutes*, a decrease in the abundance of *Bacteroidetes* (Duan et al. 2021b). The abundance of *Vibrio* significantly increases under the stress of temperature or ammonia nitrogen rising, yet decreases under simultaneous stress of temperature and ammonia nitrogen. In addition, after cold resistance variety screening, the intestinal microbiota of shrimp shows a relatively stable status in low temperature, which can alleviate the damage caused by low temperature and cold environment (Liu et al. 2019). It is concluded that when the water temperature fluctuates greatly, shrimp can strengthen its immune system by regulating the expression of temperature-related genes, so as to maintain the stability of shrimp function and repair the damaged intestinal tissue (Liu et al. 2019; Wang et al. 2020). In addition, heavy metals (sulfide, cadmium, lead, copper), nanoplastics, and aflatoxins can change the structure of shrimp intestinal microbiota (Chae et al. 2019; Duan et al. 2021a; Wang et al. 2018), which fully shows that the intestinal microbiota is an integral part of the shrimp host, and has evolved into a “multi-functional organ,” and cooperates with the shrimp host to cope with the stress of the aquaculture environment.

Besides the physical–chemical parameter, the water and sediment microbial communities also directly link to shrimp intestine microbiota. Comparing shrimp intestinal microbiota to the surrounding environment, it is found that the shared microbes account for 80%, 65%, and 77% in intestine, water, and sediment, indicating a similar microbial composition among these three habitats (Hou et al. 2018b). Moreover, water and sediment communities had a large contribution to the succession of shrimp intestine community; yet, the core communities from each habitat were distinct, suggesting that each habitat would select their core taxa. Therefore, environmental water and sediment microbial communities can shape intestine microbiota for host health, which making the environment–intestinal microbiota–host health closely relationship possible to be the central dogma in an anthropogenic aquaculture ecosystem (Huang et al. 2021). Indeed, reducing the influence or interruption of environmental microbial communities to shrimp intestinal microbiota may be a suitable way in shrimp farming. In a longitudinal study in Aquamimicry system, which uses many aerators to churn the water, the water and sediment microbiota shared fewer microbes with shrimp intestine than those in earthen ponds, shrimp farming gains a considerable income (Zeng et al. 2020), and this dissimilarity between intestine and surroundings may be a potential indictor for the healthy situation of shrimp farming.

### Diet components shaping shrimp intestinal microbiota

Shrimp itself cannot completely digest the three nutrients (carbohydrate, protein, and fat) in feed, and these nutrients can be utilized by the intestinal microbiota and produce a large number of metabolites. The dynamic balance of shrimp intestinal microbiota will adjust with different nutrient composition in feed, and cooperate with host to complete the digestion and absorption of the feed, so as to maximize the utilization of feed (Qiao et al. 2017). Carbohydrates not only provide a carbon shelf for the growth of shrimp, but also provide energy for the nutrition metabolism activities of microorganisms (Chen et al. 2021). The supplement of carbohydrates (such as glucose, xylooligosaccharides, sucrose, and starch) to the feed can improve the intestinal microbiota structure of shrimp, promote intestinal health, and improve the growth performance of shrimp (Chen et al. 2021; Gyan et al. 2022). In addition, adjusting the protein ratio or composition of feed can cause changes in the structure of shrimp intestinal microbiota, and the dynamic balance of the microbiota will be transformed in the direction conducive to the digestion of protein, so as to improve the utilization rate of feed protein (Gyan et al. 2022). Similarly, changing the proportion of oil sources in the feed (soybean oil, tallow, and linseed oil) can also achieve the same results, and further affect the immune function and growth performance of shrimp (Zhang et al. 2014). Adjustment of carbon nitrogen ratio in feed can optimize the structure of microbiota and improve intestinal nutrient metabolism (Guo et al. 2020). Further studies found that there were significant differences in the utilization of nutrients by different intestinal microbiota of shrimp: the foregut microbiota mainly metabolized amino acids and carbohydrates; intestinal microbiota mainly metabolized lipids, terpenoids, and polyketones; and the hindgut microbiota mainly metabolized cofactors, vitamins, and energy (Garibay-Valdez et al. 2021). These feed additives can directly participate in the digestion and absorption of nutrients, regulate the microecological balance of shrimp intestine, and then improve production performance and enhance the disease resistance of shrimp and increase the resistance to environmental stress by optimizing the structure of intestinal microbiota (Su et al. 2020, 2021), which can improve the probiotic function of intestinal microbiota by improving the intestinal microecological balance.

### Future perspectives

Studies of shrimp intestinal microbiota in different aquaculture conditions are increasing; more and more specific species are identified in diseased shrimp, which may be the potential biomarkers, although their exact role in causing the disease remains unclear. Consequently, further studies are necessary to establish the causal relationship between shrimp diseases and their underlying agents following “Microecological Koch’s postulates.” In addition, defining a “healthy microbiota” is an ultimate challenge in aquaculture field. As the microbial community varies with the individual, growth stage, geographic area, and many such factors, it becomes quite complicated to consider a set of microbial community as healthy. It is of great interest to identify compositional patterns across large populations and geographies to find a classification that potentiates microbiota-based diagnostics or prevention of disease. Defining a “healthy microbiota” inquires classification of distinguished markers from comparison of “diseased” and “healthy” by gathering all sequencing data in database, rather than just analyzing dozens of samples from few times or sites.

**Fig. 1:**
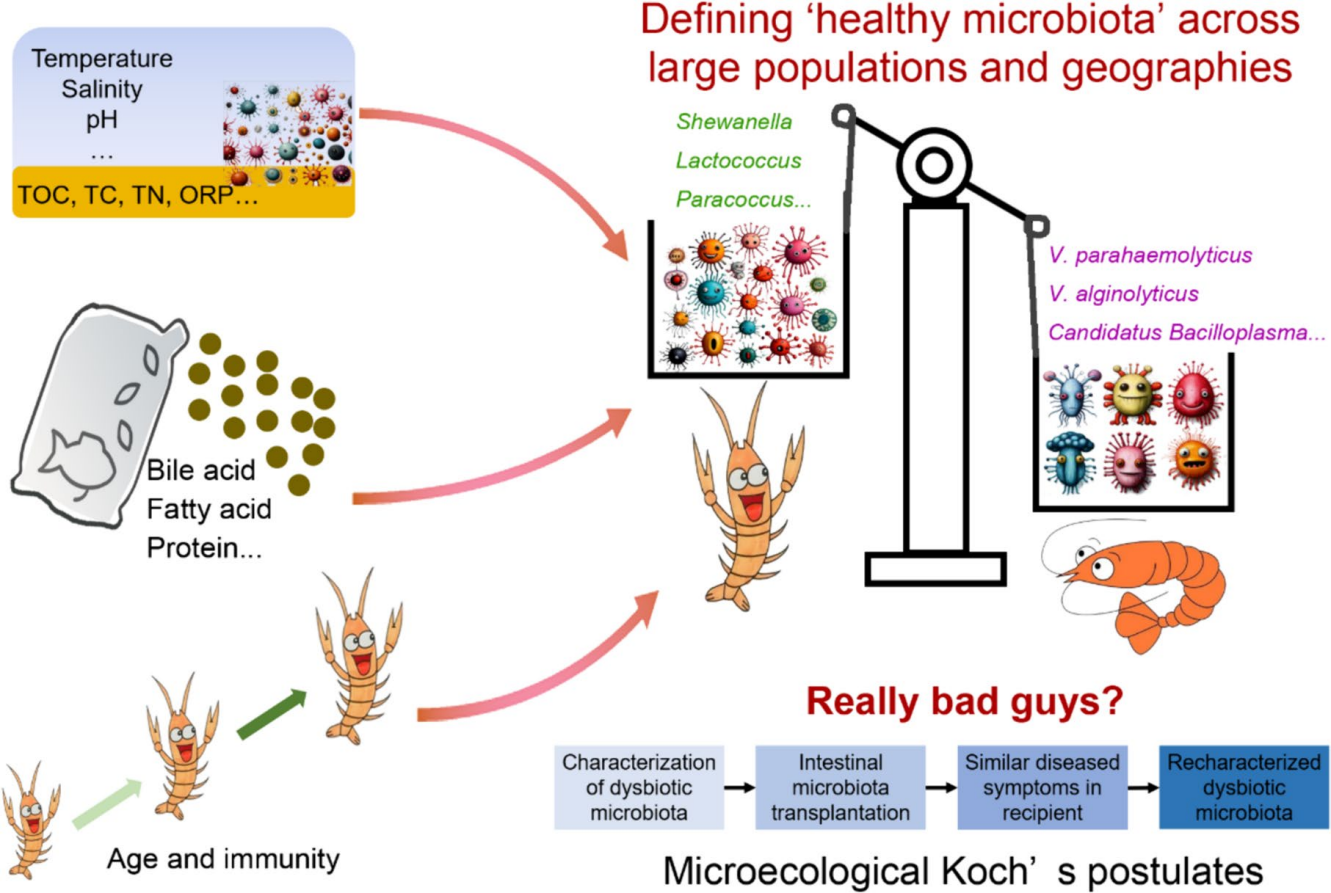
The future perspective of intestinal microbiota and shrimp culture. As shrimp intestinal microbiota is relevant to the alteration of environmental parameters/microbial communities, diets, age and immunity, the definition of “healthy microbiota” requires large data set across populations and geographies. Moreover, the microecological Koch’s postulates are the key to validate the relationship between intestinal microbiota dysbiosis and disease, which help to find the real etiology of shrimp disease

As shrimp is an invertebrate that lack specific immunity system, vaccines, or drugs are not appropriate in shrimp disease treatment. Indeed, maintaining the stability of physical and chemical factors in the aquaculture environment, reducing environmental stress factors, and using probiotics reasonably are the ways to success in shrimp farming. The use of chemicals is eliminated through the implementation of a symbiotic system, which is achieved through the application of prebiotics (non-digestible components that can be metabolized by specific microorganisms) and probiotics. Lower environmental disturbance leads to more stable shrimp intestinal composition. Collectively, the healthy aquaculture ecosystem of shrimp could be constructed based on the intestine microbiome of Microecological Koch’s postulates. The shrimp health can be promoted by improving the breeding environment and regulating the intestinal microbial community, and developing a suitable microbial micro-ecological prevention and control strategy is the main purpose of shrimp farming (Fig. 1), which can help to prevent shrimp intestinal microbiota dysbiosis and reduce the risk of disease outbreaks.
